# Working at the office or from home during the COVID-19 pandemic: a cross-sectional study of temporal patterns of sitting and non-sitting among normal-weight and overweight Brazilian office workers

**DOI:** 10.1186/s44167-023-00038-0

**Published:** 2023-12-05

**Authors:** Luiz Augusto Brusaca, David M. Hallman, Leticia Bergamin Januario, Nidhi Gupta, Ana Beatriz Oliveira, Svend Erik Mathiassen

**Affiliations:** 1 Department of Physical Therapy, Laboratory of Clinical and Occupational Kinesiology, Federal University of São Carlos, Washington Luiz Road, Km 235, SP310, São Carlos, São Paulo 13565-905 Brazil; 2 Department of Occupational Health Science and Psychology, Centre for Musculoskeletal Research, University of Gävle, 801 76 Gävle, Sweden; 3 National Research Centre for the Working Environment, Lersø Parkalle 105, 2100 Copenhagen, Denmark

**Keywords:** Occupational health, Public health, Obesity, Accelerometry, 24-h movement behaviour, Compositional data analysis

## Abstract

**Background:**

This study documents and compares temporal patterns of physical behaviours, assessed using accelerometry, on working and non-working days among normal-weight (body mass index [BMI] < 25 kg/m^2^) and overweight (BMI ≥ 25 kg/m^2^) office workers who were either working exclusively at the office (WAO) or exclusively from home (WFH) during the COVID-19 pandemic.

**Methods:**

In this cross-sectional study, behaviours were measured over 7 days using a thigh-worn accelerometer in 43 workers WAO (21 normal-weight and 22 overweight) and 73 workers WFH (33 normal-weight and 40 overweight). 24-h behaviours were completely described in terms of sitting in short (≤ 5 min), moderate (> 5 and ≤ 30 min) and long bouts (> 30 min), non-sitting in short (≤ 5 min) and long bouts (> 5 min), and time-in-bed. These behaviour compositions were transformed into five isometric log-ratios (ilr) coordinates according to compositional data analysis procedures. Differences between workplace (WAO vs. WFH) and BMI groups (normal-weight vs. overweight) were tested using ANCOVA with adjustment for age and gender.

**Results:**

Compared to workers WAO, workers WFH spent more time-in-bed relative to time awake during working days, more time sitting relative to non-sitting, less time in short bouts of sitting relative to moderate and long bouts, less time in moderate bouts of sitting relative to long bouts, and more time non-sitting in short bouts relative to long bouts. Effect sizes [$\eta_{p}^{2}$] were between 0.05 and 0.21 and *p*-values between < 0.001 and 0.04. Irrespective of workplace, overweight workers spent less time sitting in short relative to moderate and long bouts ($\eta_{p}^{2}$ = 0.06, *p* = 0.01) than normal-weight workers, while differences in the other ilr coordinates were insignificant. During non-working days, behaviours did not differ significantly by workplace, while overweight workers spent more time sitting relative to non-sitting ($\eta_{p}^{2}$ = 0.10, *p* < 0.001), less time sitting in short relative to moderate and long bouts ($\eta_{p}^{2}$ = 0.13, *p* < 0.001), and less time sitting in moderate relative to long bouts ($\eta_{p}^{2}$ = 0.04, *p* = 0.03) than normal-weight workers. We found no interactions between workplace and BMI.

**Conclusions:**

Our findings suggest that WFH and being overweight predispose to more time sitting and less temporal variation in behaviours, thus reinforcing that these workers could likely benefit from interventions to reduce prolonged sitting time and increase variation.

**Supplementary Information:**

The online version contains supplementary material available at 10.1186/s44167-023-00038-0.

## Introduction

Overweight and obesity—i.e. having a body mass index (BMI) between 25–29.9 kg/m^2^ and ≥ 30 kg/m^2^, respectively—are major public health problems affecting individuals of all ages, ethnicities and socioeconomic groups, regardless of the country they live in [1, 2]. Studies have shown that the prevalence of overweight/obesity (henceforth referred to as ‘overweight’) has increased over the years and is expected to continue increasing [2, 3]. This has lead researchers to describe overweight as a pandemic with great consequences for public health [4]. It is well known that overweight individuals are more susceptible to developing metabolic syndrome (abdominal obesity, abnormal glycemia, dyslipidaemia, and blood hypertension), increasing the risk of developing chronic non-communicable diseases (e.g., type 2 diabetes, cardiovascular diseases, neurodegenerative diseases, and cancer) [4, 5]. Overweight is also associated with more sedentary behaviour and less physical activity [6], which in its own right contributes to the development of non-communicable diseases and may lead to increased mortality [7]. Among overweight individuals, these risk factors can be mitigated if sedentary behaviour is reduced and more time is spent in physical activity [8, 9].

Since the COVID-19 outbreak, there has been a large focus on the effect on physical behaviours when individuals were, to different extents, requested to work from home. The available evidence has generally shown that working from home during the COVID-19 pandemic was associated with increased sedentary behaviour, decreased physical activity, and increased or unchanged sleep [10–14]. Despite the considerable number of studies examining behaviours during the pandemic, few studies have, to the best of our knowledge, monitored these behaviours using accelerometers [13], which are considerably more accurate than self-reports [15–17]. In particular, few studies have compared work performed either at the office or from home during the COVID-19 pandemic [18–21] and none of these studies with accelerometer measurements addressed whether normal-weight and overweight workers behaved differently, even though a few studies have addressed this issue on basis of self-reported behaviours [22, 23]. Consequently, little is known specifically about the extent to which directly measured physical behaviours differ between work at the office (WAO) and work from home (WFH) for normal-weight and overweight office workers. Furthermore, while workers’ behaviours during work days may differ by location due to different constraints when WAO and WFH [18, 24], non-working days represent a situation where workers have the same opportunities to be physically active, but where body weight may influence behaviours [25, 26]. Thus, behaviours during non-working days can be addressed to understand the extent to which possible differences at work between normal-weight and overweight individuals are due to different working conditions or are a ‘generic’ difference in behaviours.

The main focus of studies conducted during the pandemic was to investigate differences in the total volume of physical behaviours accumulated during the day [10–13], rather than investigating the temporal pattern, or variation, of physical behaviours, i.e. how behaviours are accumulated throughout the day in uninterrupted bouts of different durations [27–29]. This should not be confused with ‘temporal patterns’ related to timing of behaviours during the day, e.g., whether behaviours differ between morning and afternoon in the same day. Understanding how behaviours are accumulated is important, as some studies have suggested that breaking up prolonged sedentary behaviour in shorter periods improves markers of cardiometabolic health compared to being sedentary for longer, uninterrupted periods [30, 31]. Thus, shortening the sedentary periods may, to some extent, alleviate the negative health effects of extensive accumulated sitting [8].

In a post-pandemic “new normal” situation, WFH or combinations of WAO and WFH in a hybrid model will likely persist for a considerable part of the workforce [32, 33]. A 24-h accelerometer-based assessment of time-use among normal-weight and overweight office workers, allowing for a comprehensive understanding of temporal patterns of sitting and non-sitting (e.g., standing, moving, walking, and running) accumulated at the office and at home, is needed to provide specific behaviour recommendations for employers and policymakers, for instance regarding scheduling of posture changes and breaks from sitting. Therefore, the aim of this study was to document and compare temporal patterns of physical behaviours, assessed using accelerometry, on working and non-working days among normal-weight and overweight office workers who were working either exclusively at the office or exclusively from home during the COVID-19 pandemic. Combining evidence from before the COVID-19 pandemic with studies performed during the pandemic [11, 13, 22, 23, 25], we hypothesised that during working days, workers WAO would spend less time sitting, yet distributed in longer bouts, more time non-sitting, also distributed in longer bouts, and less time-in-bed (as a proxy for sleep) than workers WFH. We also hypothesised that normal-weight workers would spend less time sitting and more time non-sitting than overweight workers, and that time in sitting and non-sitting would be accumulated in longer bouts for overweight workers. On non-working days, we did not expect any influence on behaviours of where the workers had been working during their working days, while we still hypothesised that normal-weight workers would spend less time sitting and more time non-sitting than overweight workers, and that the latter group would accumulate time in longer bouts. We expected time-in-bed to be similar for normal-weight and overweight workers, both on working days and non-working days.

## Methods

### Study population

This cross-sectional study was conducted using data from normal-weight and overweight office workers who were working in public and private organizations in Brazil during the COVID-19 pandemic. Data were collected between September 2020 and June 2021. The criteria for inclusion of workers in the study were: (1) self-reported computer use for at least 4-h per workday; (2) engagement in office-based tasks (e.g., answering emails, data entry, processing documents, and browsing the internet); (3) employment on a full-time contract; and (4) no report of chronic health problems. Workers were invited to participate through advertisements published in the regional university’s social media.

The study was performed in accordance with the Declaration of Helsinki and approved by The Human Ethics Committee of the Federal University of São Carlos (São Carlos, SP, Brazil; registration process #50232821.3.0000.5504 and #38136420.9.0000.5504). All participants provided their written informed consent.

### Data collection

#### Demographic information

All workers meeting the inclusion criteria were asked to answer a web-based questionnaire containing demographic and personal information, including gender, age, company position (manager or employee), type of contract (permanent contract, temporary employment or fixed-term contracts), and smoking status (yes or no). Workers were also asked about household work through the question “Do you perform household work?” (yes or no), and if the answer was ‘yes’, for how many minutes per day. The location of work was assessed using a single item “where do you perform your work during the pandemic?” with three responses categories: exclusively WAO; exclusively WFH; and in a hybrid arrangement (combining WAO and WFH). No worker reported to work both at the office and from home, so the study material contained workers working exclusively at the office or exclusively from home. All respondents who answered the questionnaire were also asked if they were interested in participating in measurements of physical behaviours continuously over 7 consecutive days wearing an accelerometer. At a positive answer, the participant was contacted via e-mail or a messaging application (WhatsApp), and an in-person meeting lasting about 30 min was arranged to take place within no more than 5 days to measure anthropometry and perform the initial procedure so that physical behaviours could be assessed during the following week (see below).

#### Anthropometric measurements

Height and body mass were measured with participants wearing light clothing (e.g., t-shirt and light trousers/skirt) and being barefoot. Height was measured to the nearest 0.1 cm using a portable stadiometer (MD, Curitiba, Brazil) and body mass to the nearest 0.1 kg using a portable scale (W200 M; Welmy Balanças, Santa Bárbara d'Oeste, Brazil). Body mass index was subsequently calculated by dividing body mass by height squared (kg/m^2^). Weight status was categorised using a standardised international cut-point [1] as normal-weight (BMI < 25 kg/m^2^) or overweight (BMI ≥ 25 kg/m^2^).

#### Measurements of physical behaviours

Physical behaviours were monitored at 20 Hz in all participants using a triaxial ActivPAL Micro 4 accelerometer (PAL technologies, Glasgow, Scotland) attached to the right thigh, medially between the iliac crest and the upper border of the patella. The accelerometer was placed by a member of the research team. Due to COVID-19, three participants WFH preferred to attach the devices themselves based on written instructions and a video recorded by the researchers. During the accelerometer measurement days, participants used a diary to note their working hours, their time-in-bed (i.e., when they went to bed in the evening and the time they woke up), whether a day was off work, and the time and reason if the accelerometer was removed.

### Data processing

The accelerometer data were downloaded using the manufacturers’ software (PAL Software Suite Version 8) and processed using a custom-made MATLAB program, *Acti4* [34, 35] that classifies different behaviours (i.e., postures and activities) with a confirmed good validity.

We then identified three mutually exclusive behaviours (“compositional parts”) that completely accounted for time used during working days and non-working days separately, i.e., sitting (including lying), non-sitting (standing, moving, walking, walking stairs, running, and cycling), and time-in-bed. Sitting and non-sitting behaviours were identified based on the accelerometer data and time-in-bed was identified using the diary. Daily time spent in each behaviour was averaged over all available working days and non-working days for each worker. Only days with complete 24-h measurements were included for further analysis. Also, the working days had to contain at least 4 h of work to be included in the analysis [36, 37].

#### Exposure variation analysis

The temporal patterns of sitting and non-sitting were quantified using Exposure Variation Analysis (EVA) [27, 28, 38]. Based on the timeline of the processed accelerometer signal for working and non-working days, the occurrence of uninterrupted sitting and non-sitting periods of different durations were derived in two steps. First, we calculated a detailed EVA matrix, with uninterrupted bouts classified into six categories from ≤ 1 to > 60 min [28, 37]. In the second step we merged bout categories to avoid zeros in data, which cannot be handled in the compositional data analysis described below [39, 40]. Thus, in the second step, the behaviours were categorized as sitting in short (≤ 5 min), moderate (> 5 and ≤ 30 min) and long bouts (> 30 min); and non-sitting in short (≤ 5 min) and long bouts (> 5 min) [41, 42]. Time-in-bed was added on basis of the diary, as explained above, to arrive at a full 24-h behaviour composition. This two-step procedure is described in more detail in Additional files 1, 2.

#### Time-use compositions

Times spent in uninterrupted bouts of physical behaviours of different durations form parts of a whole and are inherently co-dependent and constrained, meaning that they share time within a finite 24-h window. Thus, more time can be spent in one behaviour only at the cost of reducing time on one or more other behaviours, so that the fixed total of 24-h, or 100%, is maintained [39, 40]. Therefore, we processed the 24-h time-use compositions according to compositional data analysis (CoDA) procedures [39, 40] using the package ‘compositions’ v2.0-2 [43] in R v4.2.0 [44], as in previous studies from our group [18–20]. Following the CoDA approach, the 24-h behaviour compositions of working and non-working days were transformed into sets of five orthogonal isometric log-ratio (ilr) coordinates [45]. This ilr transformation allows data to be analysed further using standard statistical methods [39, 40]. We considered splitting time in non-sitting into light physical activity and moderate-to-vigorous physical activity, but more than half of the workers spent zero time in moderate-to-vigorous physical activity bouts longer than 5 min, and zeroes cannot be handled in CoDA. Thus, light physical activity and moderate-to-vigorous physical activity were merged into the ‘non-sitting’ category of behaviour.

The ilr set, calculated for working and non-working days separately, specifically reflects the contrasts in behaviour that we wished to address. The five ilr-coordinates were defined as follows:

$${\mathrm{ilr}}_{1}=\sqrt{\frac{5}{6}} \mathrm{ln}\left(\frac{\text{Time-in-bed}}{\sqrt[5]{\begin{array}{c}Sitting\,in\,short\,bouts * Sitting\,in\,moderate\,bouts * Sitting\,in\,long\,bouts * \\ Non{\text{-}}sitting\,in\,short\,bouts * Non{\text{-}}sitting\,in\,long bouts\end{array}}}\right), \quad {\mathrm{ilr}}_{2}=\sqrt{\frac{6}{5}} \mathrm{ln}\left(\frac{\sqrt[3]{\mathrm{Sitting\,in\,short\,bouts }*\mathrm{ Sitting\,in\,moderate\,bouts }*\mathrm{ Sitting\,in\,long\,bouts}}}{\sqrt[2]{\mathrm{Non}-\mathrm{sitting\,in\,short\,bouts }*\text{Non-}\mathrm{sitting\,in\,long\,bouts}}}\right),$$

$${\mathrm{ilr}}_{3}=\sqrt{\frac{2}{3}} \mathrm{ln}\left(\frac{\mathrm{Sitting\,in\,short\,bouts}}{\sqrt[2]{\mathrm{Sitting\,in\,moderate\,bouts }*\mathrm{ Sitting\,in\,long\,bouts}}}\right), \quad {\mathrm{ilr}}_{4}=\sqrt{\frac{1}{2}} \mathrm{ln}\left(\frac{\mathrm{Sitting\,in\,moderate\,bouts}}{\mathrm{Sitting\,in\,long\,bouts}}\right),\quad {\mathrm{ilr}}_{5}=\sqrt{\frac{1}{2}} \mathrm{ln}\left(\frac{\text{Non-}\mathrm{sitting\,in\,short\,bouts}}{\text{Non-}\mathrm{sitting\,in\,long\,bouts}}\right),$$

ilr_1_ expresses the ratio of time-in-bed to time spent awake (i.e., all other behaviours); ilr_2_ expresses time spent sitting (all bout durations) relative to non-sitting (all bout durations); ilr_3_ expresses time spent sitting in short bouts relative to moderate and long bouts; ilr_4_ expresses time spent sitting in moderate bouts relative to long bouts; and ilr_5_ expresses time spent non-sitting in short bouts relative to long bouts.

### Statistical analysis

Characteristics of the participants were reported using frequencies and percentages for categorical data and means and standard deviation (SD) for continuous variables. Daily time spent in each behaviour averaged over all measured working days and over all non-working days for each worker were expressed in terms of compositional means, in minutes (closed to a total duration of 1440 min, i.e., 24-h) as well as percentages (closed to 100%).

The ilr-transformed data sets describing physical behaviours during working and non-working days were used to investigate the differences between the groups of workers WAO and WFH, and between normal-weight and overweight workers in an unadjusted model using two-way analysis of variance (ANOVA) with workplace (WAO vs. WFH) and BMI (normal-weight vs. overweight) entered as between-subject factors. In these five unadjusted models (one model for each of the five ilr), we first performed analysis including a two-factor interaction term between workplace and BMI, but since none of these interactions were significant, we eventually resolved the models without interaction. In a second step, we ran adjusted models for each ilr using two-way analysis of covariance (ANCOVA) controlling for gender and age. In these adjusted models, the age of the workers was centred on the mean age of the population. In all analysis, partial eta squared ($\eta_{p}^{2}$) was used as a measure of effect size, and the corresponding *p*-value as a complementary metric for evaluating statistical significance. Small, medium, and large effects were categorized using discrimination values of $\eta_{p}^{2}$ of 0.01, 0.06, and 0.14 [46]. All statistical analysis were carried out using the software R v4.2.0 [44].

## Results

### Flow of participants

**Fig. 1:**
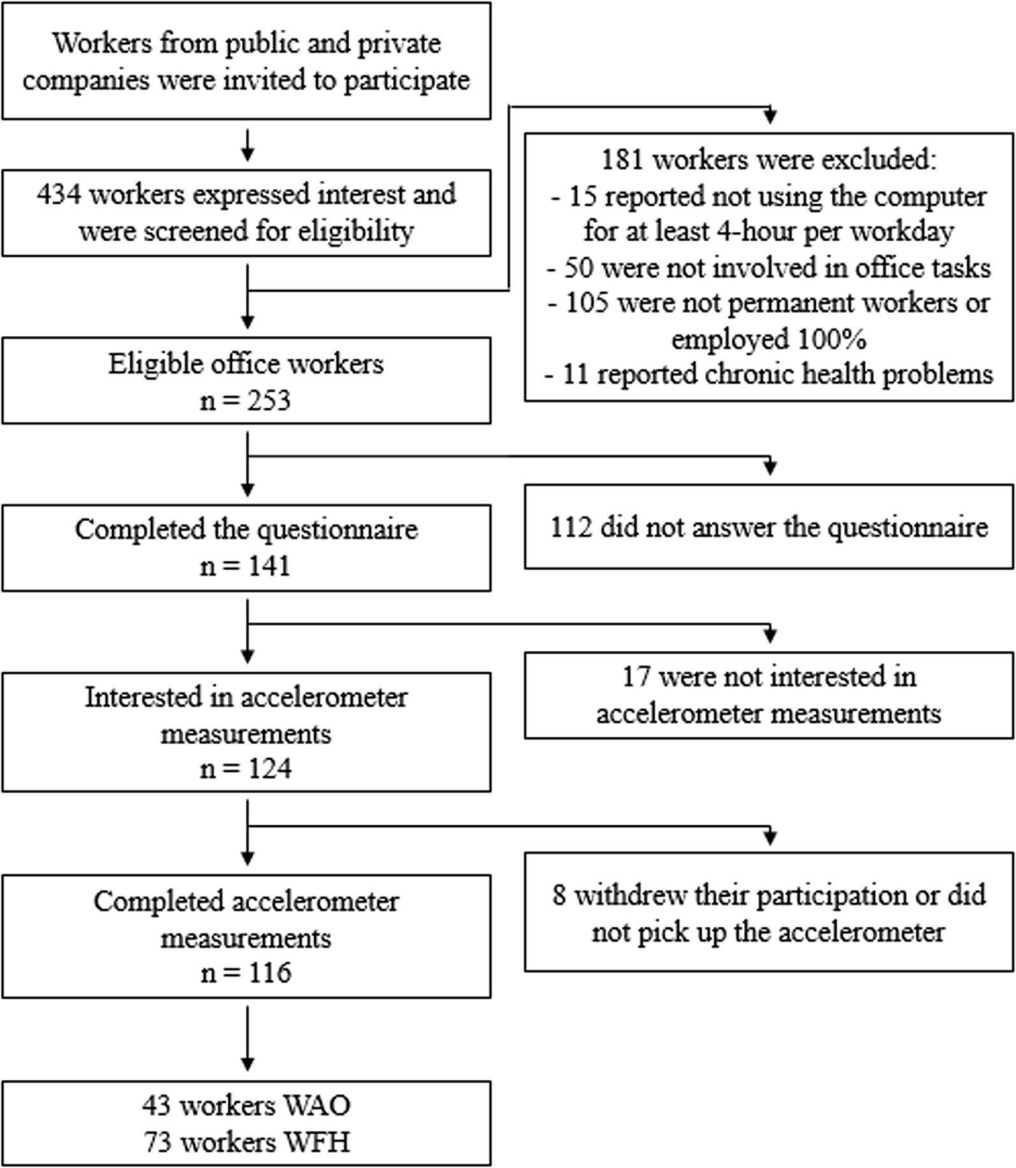
Flow chart of participant recruitment and data collection. *WAO* working at the office, *WFH* working from home

The flow of participants is illustrated in Fig. 1. Of a total of 434 office workers who expressed interest in participating, 253 met the inclusion criteria, and 141 completed the questionnaire (response rate 56%; Fig. 1). One hundred and sixteen of these took part in the accelerometer measurements. Among these 116 workers, 43 worked exclusively at the office and 73 exclusively from home. During the 10-month recruitment period between September 2020 and June 2021, participants in the four groups (i.e., WAO and WFH, normal-weight and overweight) entered the study in a relatively similar way.

### Characteristics of the study population

In the WAO group, the number of women (*n* = 21) and men (*n* = 22) was balanced (‘All workers’, Table 1), while workers WFH included slightly more women (*n* = 39) than men (*n* = 34). On average, workers WAO were older (39.3 years; SD 9.3) than workers WFH (33.5 years; SD 9.2). Workers WAO had more often a management position in the company compared with workers WFH. No marked differences were observed in smoking, household work, and BMI between workers WAO and WFH (‘All workers’, Table 1). Normal-weight and overweight workers WAO appeared to differ slightly in gender, company position, and household work (Table 1), while the two WFH groups appeared to differ in household work. Of note, no underweight (BMI < 18.5 kg/m^2^) workers were included in the normal-weight group.

**Table 1:** Demographic and social characteristics of participants with accelerometry measurements. Descriptive results are presented for workers working at the office (WAO) and from home (WFH) and stratified by normal-weight (body mass index [BMI] < 25 kg/m^2^) and  overweight (BMI ≥ 25 kg/m^2^) workers in each location

	Work at the office (WAO)			Work from home (WFH)		
	All workers *n* = 43	BMI < 25 *n* = 21	BMI ≥ 25 *n* = 22	All workers n = 73	BMI < 25 *n* = 33	BMI ≥ 25 *n* = 40
Gender, n (%)^a^						
Women	21 (48.8)	12 (57.1)	9 (40.9)	39 (53.4)	18 (54.5)	21 (52.5)
Men	22 (51.2)	9 (42.9)	13 (59.1)	34 (46.6)	15 (45.5)	19 (47.5)
Age (years), mean (SD)^a^	39.3 (9.3)	38.1 (9.3)	40.5 (9.4)	33.5 (9.2)	33.2 (9.0)	33.7 (9.4)
Company position, n (%)^a^						
Manager	15 (34.9)	6 (28.6)	9 (40.9)	5 (6.8)	2 (6.1)	3 (7.5)
Employee	28 (65.1)	15 (71.4)	13 (59.1)	68 (93.2)	31 (93.9)	37 (92.5)
Smokers (yes), n (%)^a^	4 (9.3)	3 (14.3)	1 (4.5)	3 (4.1)	1 (3.0)	2 (5.0)
Household work^a^						
Perform (yes), n (%)	32 (74.4)	16 (76.2)	16 (72.7)	59 (80.8)	30 (90.9)	29 (72.5)
Minutes per day, mean (SD)	56.7 (46.7)	66.2 (55.5)	47.7 (35.4)	65.0 (48.4)	71.4 (42.7)	59.8 (52.6)
BMI (kg/m^2^), mean (SD)^b^	26.5 (5.1)	22.7 (1.7)	30.2 (4.5)	26.8 (4.8)	22.5 (1.7)	30.3 (3.6)
Accelerometer data^b^						
Total hours recorded	5328	2568	2760	8592	3984	4608
Days per worker, mean (SD)	5.2 (0.4)	5.1 (0.3)	5.2 (0.5)	4.9 (0.7)	5.0 (0.3)	4.8 (0.9)
Hours per worker, mean (SD)	123.9 (10.4)	122.3 (7.2)	125.5 (12.7)	117.7 (17.5)	120.7 (7.3)	115.2 (22.5)

^a^Self-reported information from online questionnaire

^b^Directly measured

Filtering the data to only include days with complete 24-h measurements and, for working days, at least 4 h of work, resulted in a total of 5328 h (222 days) of accelerometer recording for workers WAO and 8592 h (358 days) for workers WFH, respectively, with, on average, 123.9 h (SD 10.4) and 117.7 h (SD 17.5) of data per worker. The average number of days collected from workers WAO was 5.2 (SD 0.4) and from workers WFH 4.9 (SD 0.7). The corresponding values for normal-weight and overweight workers are presented in Table 1.

### Physical behaviour compositions

The compositional mean values of time spent sitting, non-sitting and in bed during working and non-working days of office workers WAO and WFH are shown in Table 2. During working days, workers WAO appeared to spend less time sitting, more time non-sitting and less time-in-bed than workers WFH. On non-working days, workers WAO appeared to spend less time sitting and more time non-sitting than workers WFH; while time spent in bed appeared to differ only to a minor extent between workers WAO and WFH (Table 2).

**Table 2:** Compositional means (SD between workers) in minutes per day and in percent, of sitting, non-sitting and time-in-bed for office workers working at the office (WAO) and from home (WFH) during working and non-working days. Within the WAO and WFH groups, data are shown for normal-weight (body mass index [BMI] < 25 kg/m^2^) and overweight (BMI ≥ 25 kg/m^2^) workers

	Behaviours	Working days			Non-working days		
		All workers	BMI < 25	BMI ≥ 25	All workers	BMI < 25	BMI ≥ 25
Work at the office (WAO)							
Minutes	Sitting short	52.2 (22.4)	58.2 (26.4)	46.4 (16.3)	50.7 (21.2)	58.3 (24.1)	43.4 (15.2)
	Sitting moderate	304.2 (83.3)	308.2 (88.1)	300.3 (80.4)	220.7 (65.3)	201.8 (57.5)	238.7 (68.5)
	Sitting long	352.9 (131.7)	320.6 (125.2)	383.7 (133.2)	301.7 (113.7)	280.1 (113.3)	322.3 (112.9)
	Non-sitting short	80.7 (22.7)	87.8 (22.0)	74.0 (21.8)	72.9 (31.2)	85.0 (34.2)	61.4 (23.3)
	Non-sitting long	211.3 (66.0)	218.7 (63.1)	204.3 (69.3)	275.8 (112.6)	293.7 (119.4)	258.8 (105.7)
	Time-in-bed	438.7 (56.0)	446.4 (59.8)	431.3 (52.5)	518.2 (81.7)	521.0 (79.2)	515.4 (85.8)
Percentage	Sitting short	3.6 (1.6)	4.0 (1.8)	3.2 (1.1)	3.5 (1.5)	4.1 (1.7)	3.0 (1.1)
	Sitting moderate	21.1 (5.8)	21.4 (6.1)	20.9 (5.6)	15.3 (4.5)	14.0 (4.0)	16.6 (4.8)
	Sitting long	24.5 (9.1)	22.3 (8.7)	26.6 (9.3)	20.9 (7.9)	19.5 (7.9)	22.4 (7.8)
	Non-sitting short	5.6 (1.6)	6.1 (1.5)	5.1 (1.5)	5.1 (2.2)	5.9 (2.4)	4.3 (1.6)
	Non-sitting long	14.7 (4.6)	15.2 (4.4)	14.2 (4.8)	19.2 (7.8)	20.4 (8.3)	18.0 (7.3)
	Time-in-bed	30.5 (3.9)	31.0 (4.2)	30.0 (3.6)	36.0 (5.7)	36.2 (5.5)	35.8 (6.0)
Work from home (WFH)							
Minutes	Sitting short	45.4 (20.4)	50.1 (21.4)	41.6 (19.1)	45.6 (18.0)	51.0 (18.0)	40.8 (16.9)
	Sitting moderate	245.5 (75.0)	242.2 (77.9)	248.2 (73.5)	231.6 (71.5)	243.0 (73.5)	221.4 (69.1)
	Sitting long	478.4 (142.6)	462.6 (148.0)	491.1 (138.8)	358.9 (153.4)	308.5 (145.5)	403.8 (147.9)
	Non-sitting short	66.4 (22.3)	72.5 (24.5)	61.6 (19.4)	68.8 (23.8)	78.6 (24.8)	60.0 (19.4)
	Non-sitting long	139.0 (72.6)	140.9 (72.1)	137.4 (73.9)	228.4 (102.0)	249.3 (90.7)	209.7 (108.9)
	Time-in-bed	465.2 (57.7)	471.7 (58.7)	460.0 (57.0)	506.8 (75.4)	509.7 (74.3)	504.3 (77.2)
Percentage	Sitting short	3.2 (1.4)	3.5 (1.5)	2.9 (1.3)	3.2 (1.3)	3.5 (1.3)	2.8 (1.2)
	Sitting moderate	17.1 (5.2)	16.8 (5.4)	17.2 (5.1)	16.1 (5.0)	16.9 (5.1)	15.4 (4.8)
	Sitting long	33.2 (9.9)	32.1 (10.3)	34.1 (9.6)	24.9 (10.7)	21.4 (10.1)	28.0 (10.3)
	Non-sitting short	4.6 (1.6)	5.0 (1.7)	4.3 (1.3)	4.8 (1.7)	5.5 (1.7)	4.2 (1.3)
	Non-sitting long	9.7 (5.0)	9.8 (5.0)	9.5 (5.1)	15.9 (7.1)	17.3 (6.3)	14.6 (7.6)
	Time-in-bed	32.3 (4.0)	32.8 (4.1)	32.0 (4.0)	35.2 (5.2)	35.4 (5.2)	35.0 (5.4)

Normal-weight workers WAO or WFH appeared to spend less time sitting and more time non-sitting than overweight workers, both during working and non-working days (Table 2). The normal-weight workers also appeared to have more temporal variation in their sitting and non-sitting behaviours than the overweight workers, i.e., they spent more time sitting in short and moderate bouts and in short bouts of non-sitting.

### Physical behaviour compositions expressed as ilr-coordinates

Table 3 shows the ilr-coordinates for office workers WAO and WFH in a format corresponding to Table 2; i.e., for normal-weight and overweight workers in each location. These ilr-coordinates contain the same information as the absolute durations of behaviours shown in Table 2, but now in relative terms, and transformed according to CoDA procedures as described above. Thus, a straightforward comparison is not justified.

**Table 3:** Mean (with SD between workers) of the isometric log-ratio (ilr) coordinates of office workers working at the office (WAO) and from home (WFH) during working and non-working days. Within the WAO and WFH groups, data are shown for normal-weight (body mass index [BMI] < 25 kg/m^2^) and overweight (BMI ≥ 25 kg/m^2^) workers

ilr-coordinates	Working days			Non-working days		
	All workers	BMI < 25	BMI ≥ 25	All workers	BMI < 25	BMI ≥ 25
Work at the office (WAO)						
ilr_1_: time-in-bed/awake	0.98 (0.18)	0.97 (0.20)	0.99 (0.16)	1.21 (0.27)	1.19 (0.26)	1.24 (0.27)
ilr_2_: sitting/non-sitting	0.32 (0.25)	0.25 (0.24)	0.38 (0.25)	0.08 (0.43)	– 0.06 (0.50)	0.21 (0.32)
ilr_3_: sitting short/moderate + long	– 1.51 (0.38)	– 1.40 (0.40)	– 1.63 (0.33)	– 1.34 (0.48)	– 1.15 (0.50)	– 1.53 (0.38)
ilr_4_: sitting moderate/long	– 0.08 (0.46)	– 0.01 (0.49)	– 0.15 (0.43)	– 0.18 (0.40)	– 0.17 (0.42)	– 0.19 (0.39)
ilr_5_: non-sitting short/long	– 0.67 (0.31)	– 0.64 (0.27)	– 0.70 (0.35)	– 0.94 (0.44)	– 0.86 (0.43)	– 1.01 (0.44)
Work from home (WFH)						
ilr_1_: time-in-bed/awake	1.19 (0.26)	1.17 (0.30)	1.20 (0.24)	1.22 (0.26)	1.18 (0.25)	1.26 (0.28)
ilr_2_: sitting/non-sitting	0.70 (0.45)	0.66 (0.46)	0.73 (0.45)	0.24 (0.41)	0.10 (0.38)	0.36 (0.40)
ilr_3_: sitting short/moderate + long	– 1.69 (0.43)	– 1.59 (0.41)	– 1.77 (0.43)	– 1.51 (0.44)	– 1.37 (0.44)	– 1.64 (0.40)
ilr_4_: sitting moderate/long	– 0.46 (0.47)	– 0.46 (0.45)	– 0.46 (0.49)	– 0.27 (0.50)	– 0.12 (0.49)	– 0.41 (0.48)
ilr_5_: non-sitting short/long	– 0.44 (0.46)	– 0.39 (0.43)	– 0.47 (0.49)	– 0.82 (0.38)	– 0.80 (0.33)	– 0.83 (0.43)

A positive ilr shows that time spent in the numerator behaviour was larger than time spent in the denominator behaviour, and vice versa if the ilr value is negative

### Statistical analysis of physical behaviour compositions expressed as ilr-coordinates

Table 4 shows the results of the analysis of adjusted models controlling for gender and age. Overall, the effect size in the adjusted models either increased slightly or remained unchanged, compared to the unadjusted models. The adjusted models showed statistically significant main effects of workplace (WAO vs. WFH) for all ilr-coordinates during working days (Table 4), with office workers WAO spending less time-in-bed relative to all other behaviours (ilr_1_), less time sitting in all bouts relative to non-sitting in all bouts (ilr_2_), more time in short bouts of sitting relative to moderate and long bouts (ilr_3_), more time in moderate bouts of sitting relative to long bouts (ilr_4_), and less time non-sitting in short bouts relative to long bouts (ilr_5_), compared with workers WFH. These results are consistent with the descriptive information reported in Table 3. Irrespective of workplace, normal-weight workers spent significantly more time sitting in short bouts relative to moderate and long bouts during working days (ilr_3_) compared with overweight workers, but no other significant differences were found (Table 4). We found statistically significant differences in ilr_3_ between genders, and in ilr_1_, ilr_2_, ilr_3,_ and ilr_5_ for age (cf. Additional file 3).

**Table 4:** Effects of workplace (work at the office [WAO] vs. work from home [WFH]) and body mass index (BMI; normal-weight [BMI < 25 kg/m^2^] vs. overweight [BMI ≥ 25 kg/m^2^]) for each isometric log-ratio (ilr) coordinate. The table shows effect size (partial eta squared, $\eta_{p}^{2}$ ), F-statistics, and p-value

ilr-coordinates	Workplace: WAO vs*.* WFH			BMI: < 25 vs*.* ≥ 25		
	$\eta_{p}^{2}$	F	*p*-value	$\eta_{p}^{2}$	F	*p*-value
Working days						
ilr_1_: time-in-bed/awake	**0.16**	**18.50**	** < 0.001**	< 0.01	0.54	0.47
ilr_2_: sitting/non-sitting	**0.21**	**25.14**	** < 0.001**	0.02	2.08	0.15
ilr_3_: sitting short/moderate + long	**0.05**	**4.30**	**0.04**	**0.06**	**7.08**	**0.01**
ilr_4_: sitting moderate/long	**0.14**	**17.29**	** < 0.001**	< 0.01	0.38	0.54
ilr_5_: non-sitting short/long	**0.08**	**8.24**	**0.005**	0.01	0.83	0.37
Non-working days						
ilr_1_: time-in-bed/awake	< 0.01	0.01	0.94	0.02	2.66	0.11
ilr_2_: sitting/non-sitting	0.04	3.75	0.06	**0.10**	**12.29**	** < 0.001**
ilr_3_: sitting short/moderate + long	0.04	3.29	0.07	**0.13**	**16.18**	** < 0.001**
ilr_4_: sitting moderate/long	0.01	0.80	0.37	**0.04**	**4.76**	**0.03**
ilr_5_: non-sitting short/long	0.02	2.13	0.15	0.01	1.01	0.32

The ANCOVA models treated workplace (WAO vs. WFH) and BMI (normal-weight vs. overweight) as between-subjects factors. Models were adjusted for gender and age. Results with *p* < 0.05 are shown in bold

On non-working days, we did not find any significant difference between the office workers WAO and WFH for any ilr-coordinate, while normal-weight workers differed significantly from overweight workers in ilr_2_, ilr_3_ and ilr_4_ (Table 4). Thus, compared to normal-weight workers, overweight workers spent more time sitting relative to non-sitting (ilr_2_; Table 3) during non-working days, less time sitting in short bouts relative to moderate and long bouts (ilr_3_), and less time sitting in moderate bouts relative to long bouts (ilr_4_). We also found a statistically significant difference in ilr_2_ and ilr_3_ for age (cf. Additional file 3).

## Discussion

In the present study, we documented and compared temporal patterns of sitting (including lying), non-sitting and time-in-bed (as a proxy for sleep) based on accelerometer recordings on working and non-working days during the COVID-19 pandemic for normal-weight and overweight office workers who were working either exclusively at the office or exclusively from home.

Our results indicated that during working days, differences in behaviours were mainly explained by the workplace where work was performed, i.e., at the office or from home (Table 4), confirming our hypothesis that workers WAO would spend less time sitting, accumulated in longer bouts, more time non-sitting, also accumulated in longer bouts, and less time-in-bed than workers WAO. Our findings agree with recent systematic reviews reporting that WFH during the COVID-19 pandemic was associated with increased sedentary behaviour, decreased physical activity, and increased time-in-bed [10–13]. Adding to the results of these systematic reviews, a large longitudinal study during the pandemic based on 17 rounds of self-reported data showed that workers WFH were more likely to be sedentary than workers WAO [14]. Our finding that workers WFH spent more time sitting in long uninterrupted bouts than workers WAO was also reported by one study using accelerometry [47] and another study using self-reports [48]. These studies found that WFH was even associated with less time spent in physical activity compared with WAO. Furthermore, and partially corroborating our results, a recent study based on accelerometry showed that office workers WFH spent more time sitting than office workers WAO, while time spent standing and stepping (i.e. walking) was similar for WFH and WAO [21]. One possible explanation for the different behaviours among workers WFH compared to workers WAO may be that commuting is not needed to the same extent when WFH, and that walking around the workplace to talk to co-workers does not take place anymore [47]. The accelerometry study by Hallman et al. [18] on workers in hybrid arrangements showed that the relative distribution of physical behaviours during time awake did not differ significantly between days WFH and days WAO, while the proportion of time spent in bed relative to time awake was larger on days WFH than on days WAO. Similar results were also observed in studies developed before [24] and during [14] the pandemic. Still, caution should be made when comparing these findings with ours, for instance since workers in high-income countries were used to WFH even before the pandemic [49].

Regarding the effects of body weight, we did not find any significant difference during working days in the ratio of total sitting to total non-sitting (ilr_2_, Table 4), contrary to our expectation that overweight workers would spend more time sitting and less time non-sitting than normal-weight workers. We only found that overweight workers spent less time sitting in short bouts relative to moderate and long bouts compared with normal-weight workers (ilr_3_), which indicates a less variable behaviour. To our knowledge, no previous study has compared the behaviours of normal-weight and overweight office workers on working days during the pandemic, neither using accelerometers nor using self-reported measures. Notwithstanding, our findings disagree with a systematic review Silveira et al. [6] published before the pandemic, suggesting that overweight is generally associated with increased sedentary time and reduced physical activity. A possible explanation is that the review included data from the general population and did not differentiate between working and non-working days. Also, the review included studies based both on accelerometry and on self-reports, and self-reported sitting is known to be biased [15]. Other studies of office workers conducted before the pandemic show conflicting results. For instance, the accelerometry study by Hadgraft et al. [26] observed less total and less prolonged (i.e., in uninterrupted periods ≥ 30 min) time sitting at the workplace in overweight and obese office workers than in normal-weight workers, while the study by Clemes et al. [25] observed that obese office workers self-reported a larger total daily sitting time than normal-weight and overweight workers during working days.

During non-working days, we did not find any difference in the behaviours of office workers according to the place where they worked, agreeing with our hypothesis that the location of work would not influence behaviours during non-working days. On the other hand, we found that body weight may have influenced physical behaviours more during non-working days than during working days. Our findings indicated that overweight workers spent more time sitting in total, relative to non-sitting in total, than normal-weight workers (ilr_2_, Table 4). Since time-in-bed was similar in the two groups, this confirmed our expectation of overweight workers spending more time sitting than normal-weight workers. We also observed that overweight workers had less temporal variation in their sitting behaviours than normal-weight workers, i.e., they spent less time sitting in short and moderate uninterrupted bouts (ilr_3_ and ilr_4_). To our knowledge, studies during the pandemic comparing behaviours between normal-weight and overweight workers are scarce. Only two studies have reported data related to weekly behaviours and leisure-time physical activity according to weight status. Specifically, the study by Giustino et al. [22] showed that overweight individuals had less self-reported physical activity compared with underweight and normal-weight individuals during the pandemic. Corroborating this, the self-report study by Moura et al. [23] showed that overweight individuals were more likely to be physically inactive than normal-weight individuals before and during the pandemic. Additionally, the study by Clemes et al. [25] reported data also from non-working days, showing that obese office workers self-reported a larger total daily sitting time than normal-weight and overweight workers even during non-working days, and that the difference between weight groups were larger during non-working days than during working days. The fact that overweight and normal-weight workers did not differ on working days but did so on non-working days, indicates that there was a basic difference in behaviours, but that it was so small that it could be overruled during working days, likely by the (not weight dependent) task demands posed on everybody at work.

### Recommendations

Our key result is that sitting time is larger, relative to non-sitting, among workers exclusively WFH than among those exclusively WAO. However, regardless of the location of work, the studied Brazilian office workers spent more than 40% of their day sitting (i.e., about 9.6 h per day). International recommendations estimate that 8 h or more of sitting per day (i.e., at least 33% of the day) leads to increased health risks [50, 51]. This can generate an extensive economic burden for society [4, 7, 52]. Increasing physical activity of any intensity and breaking up sitting as often as possible (i.e., into shorter bouts) may be a way to counteract or mitigate the health risks caused by extensive sitting [8, 9, 30, 31], and thus aid in reducing the economic burden [53]. Furthermore, sleeping too much or too little may also lead to health problems, while a sleep duration of 7–8 h per night is positively associated with health outcomes [54]. We observed, based on workers’ self-reports, that during working days around 51% of workers WAO (10 normal-weight and 12 overweight) and 42% of workers WFH (14 normal-weight and 16 overweight) spent between 7 and 8 h per day in bed, while on non-working days corresponding numbers were 16% (2 normal-weight and 5 overweight) and 31% (14 normal-weight and 8 overweight), respectively. On working days, most normal-weight and overweight workers WAO who did not fall in the 7–8 h interval spent less than 7 h in bed, while workers WFH typically spent more than 8 h per day in bed. During non-working days, most workers spent more than 8 h per day in bed. Thus, our results suggest that at least some workers need to be encouraged to sleep 7–8 h per day. Still, our findings related to time-in-bed (as a proxy for sleep) should be interpreted with caution, since this information was self-reported in the diary and may be biased to some extent.

As daily behaviours are inherently co-dependent and constrained because they share time within a finite 24-h window [39, 40], future interventions should emphasise the importance of addressing behaviours in the entire 24-h perspective. This is even emphasized in a number of 24-h movement guidelines [55–58].

### The post-pandemic “new normal” situation

The COVID-19 pandemic changed the daily life of the entire population around the world. Many of the changes caused by the pandemic have remained even after the pandemic, in particular among office workers who will WFH or in hybrid arrangements to a larger extent than before the pandemic [32, 59]. At that time, hybrid work arrangements were relatively uncommon in many countries (mainly low- and middle-income countries), and workers were not prepared to work from home or in a hybrid arrangement when the pandemic came [59, 60]. This lack of preparedness may have led to larger effects on physical behaviours in terms of, e.g., temporal variation and time-in-bed, among office workers who were not used to performing work from home [19, 20] than among those who were already used to work in hybrid arrangements [14, 18, 21]. Cultural and socioeconomic differences between countries may also have affected the post-pandemic extent of implementation of WFH and hybrid arrangements [61, 62]. Thus, it is important to understand that the future development of WFH and hybrid work will not be uniform across countries.

The preference of where to work (e.g., WAO, WFH or in a hybrid arrangement) may influence physical behaviours, in an analogy to psychological distress [63]. Thus, preference is likely an important factor, as it gives workers the opportunity to adapt routines according to personal needs [60]. As the pandemic has likely created a “new normal” in the occupational field, it is important to conduct research that can aid in developing hybrid arrangements, which are both adapted to each individual’s preferences and socioeconomic situation, and to the employer’s need for workers being present at the working place.

Finally, it remains to be seen in which direction this post-pandemic scenario will influence the health of normal-weight and overweight workers, including whether the two groups will differ in their response to hybrid arrangements. Workers’ health has been shown to have deteriorated during the pandemic [10–13, 64], but it is possible that the flexibility created by this “new normal”, along with access to community resources (e.g., parks, playgrounds, walking trails), gyms, and sport facilities, may create opportunities to promote health that were not present to the same extent before the pandemic.

### Strengths and limitations

The main strength of the present study is the use of accelerometer-based measurements both during working and non-working days to identify temporal patterns in a complete 24-h array of physical behaviours, using the *Acti4* program to identify these behaviours with a confirmed good validity [34, 35]. Another major strength is the use of a CoDA approach to process the data, which adequately handles the compositional structure of time-use data of physical behaviours [39, 40].

A limitation of the study was that associations found in the results do not imply causation, since it is impossible to evaluate causality in a cross-sectional design [65]. Therefore, the results may have been influenced by reverse causation, for instance in that, the association between body weight and physical behaviours may be in both causal directions: more physical activity may lead to less overweight and less overweight may lead to more physical activity. Also, it is not possible to determine whether behaviours on non-working days had an effect on behaviours during working days, or vice-versa. Another limitation was that we included only office workers who were working either exclusively at the office or exclusively from home. This may limit the relevance of our results for workers in a hybrid model, which seems to be a common work arrangement in post-pandemic times, as discussed above. Investigating these two independent groups also leads to a less effective statistical design than had the same workers worked at the office and from home on different days in a repeated measures design. The risk of confounding also increases in a design comparing two independent groups. For instance, workers WFH appear to be younger than workers WAO (cf. Table 1), and since age is known to influence physical behaviours, the difference between WFH vs. WAO may, to some extent, be confounded by age. Data collection had to be performed on a convenience sample that was not random, and this may have limited the generalizability of our results. We also did not calculate the sample size a priori, although we likely had sufficient power to detect most of the hypothesized effects, as shown, e.g., in Table 4. Another limitation is that we collected data at different time points (between September 2020 and June 2021). This may have influenced behaviours since the pandemic had different phases over time. However, since workers both WAO and WFH were included in parallel during the data collection period, we believe this to be a minor issue. In addition, Brazil had recommendations for social isolation during the pandemic that were voluntary and relied largely on individual responsibility. We did not, however, have access to information on the extent to which workers followed these public health recommendations at the time of data collection. Additional variables, addressing, e.g., socioeconomic status and household characteristics, could have helped to better understand the results found in our study. Notwithstanding these limitations, our study provide evidence about the 24-h time-use compositions of physical behaviours among normal-weight and overweight office workers WAO and WFH during the pandemic, which may be used by public health policy-makers from low- and middle-income countries with a similar socioeconomic status as Brazil [66].

## Conclusions

We found that during working days, workers exclusively working from home spent more time-in-bed relative to time awake, more time sitting in total relative to non-sitting in total, less time in short bouts of sitting relative to moderate and long bouts, less time in moderate bouts of sitting relative to long bouts, and more time non-sitting in short bouts relative to long bouts compared with workers exclusively working at the office. Also, we observed that overweight workers spent less time sitting in short bouts than in longer uninterrupted bouts compared with normal-weight workers. On non-working days, the workplace did not modify the physical behaviours, while workers’ weight status did. Specifically, during non-working days overweight workers spent more time sitting than non-sitting, less time sitting in short than moderate and long bouts, and less time sitting in moderate than long bouts compared with normal-weight workers. The office workers in the present study, especially workers working from home and overweight workers, spent extensive amount of time sitting, predominantly in uninterrupted bouts longer than 30 min, and they could likely benefit from interventions to reduce prolonged sitting time.

## Supplementary Information

**Additional file 1:** Steps to derive the uninterrupted bouts of behaviours used in the study.

**Additional file 2:** Compositional means of physical behaviours of office workers stratified by gender and age (table S1), mean of the corresponding isometric log-ratio coordinates (Table S2), and the effects of gender and age adjustment variables on each isometric log-ratio (ilr) coordinate (Table S3).

**Additional file 3:** STROBE Statement—checklist of items that should be included in reports of cross-sectional studies.

## Data Availability

The dataset generated and/or analysed during the current study is not publicly available due to confidentiality of some data sources, but processed data is available from the corresponding author on reasonable request.

## Abbreviations

BMI: Body mass index
COVID-19: Coronavirus disease 2019
WAO: Work at the office
WFH: Work from home
EVA: Exposure variation analysis
CoDA: Compositional data analysis
ilr: Isometric log-ratio
SD: Standard deviation
ANOVA: Analysis of variance
ANCOVA: Analysis of covariance
