# Supplementary material for: Working at the office or from home during the COVID-19 pandemic: a cross-sectional study of temporal patterns of sitting and non-sitting among normal-weight and overweight Brazilian office workers
Source: J Act Sedentary Sleep Behav. 2023 Dec 5;2:28. doi: 10.1186/s44167-023-00038-0 (PMC11960272; doi:10.1186/s44167-023-00038-0)
Supplement: Supplementary file 1 — Additional file 1: Steps to derive the uninterrupted bouts of behaviours used in the study. [file 44167_2023_38_MOESM1_ESM.docx]

**Additional file 1**

**Additional file 1.** Steps to derive the uninterrupted bouts of behaviours used in the study.

In the first step, we determined the temporal pattern of sitting and non-sitting, for working days and non-working days, according to a detailed Exposure Variation Analysis (EVA) matrix with six categories of uninterrupted bouts (≤1 min, >1 and ≤5 min, >5 and ≤10 min, >10 and ≤30 min, >30 and ≤60 min, and >60 min). The results of behaviours in minutes from this detailed EVA matrix are shown in Table S1.

| **Table S1.** Means (with SD across workers) in minutes of the Exposure Variation Analysis matrix of different uninterrupted bouts (ranging from ≤1 to >60 min) of sitting and non-sitting behaviour, and time-in-bed of office workers working at the office (WAO) and from home (WFH) during working days and non-working days. Within the WAO and WFH groups, data are shown for normal-weight (body mass index [BMI] <25 kg/m^2^) and overweight (BMI ≥25 kg/m^2^) workers. | | | | | | | |
| --- | --- | --- | --- | --- | --- | --- | --- |
| **Work at the office (WAO)** | | | | | | | |
| Behaviours  and bouts | | Working days | | | Non-working days | | |
|  |  | All workers | BMI <25 | BMI ≥25 | All workers | BMI <25 | BMI ≥25 |
| Sitting | ≤1 | 8.8 (4.0) | 11.0 (4.5) | 6.8 (2.0) | 9.6 (6.3) | 12.9 (7.1) | 6.4 (2.9) |
|  | >1 and ≤5 | 43.3 (19.7) | 47.2 (23.0) | 39.7 (15.5) | 41.1 (17.3) | 45.4 (20.0) | 37.1 (13.5) |
|  | >5 and ≤10 | 70.4 (25.8) | 74.5 (27.8) | 66.4 (23.8) | 59.6 (25.7) | 62.8 (24.0) | 56.5 (27.4) |
|  | >10 and ≤30 | 233.8 (67.4) | 233.8 (73.1) | 233.9 (63.2) | 161.1 (52.4) | 139.0 (47.4) | 182.1 (49.1) |
|  | >30 and ≤60 | 186.0 (71.0) | 168.8 (64.5) | 202.5 (74.4) | 134.0 (53.5) | 119.7 (54.1) | 147.6 (50.3) |
|  | >60 | 166.9 (110.5) | 151.8 (100.4) | 181.2 (119.8) | 167.7 (90.1) | 160.4 (86.5) | 174.7 (94.9) |
| Non-sitting | ≤1 | 11.6 (5.5) | 13.5 (6.6) | 9.7 (3.3) | 11.2 (5.8) | 13.0 (6.9) | 9.4 (3.9) |
|  | >1 and ≤5 | 69.2 (19.1) | 74.3 (18.3) | 64.3 (19.0) | 61.8 (26.3) | 72.0 (28.6) | 52.0 (20.0) |
|  | >5 and ≤10 | 64.4 (18.8) | 70.6 (21.3) | 58.5 (14.2) | 61.2 (22.0) | 69.3 (23.5) | 53.4 (17.8) |
|  | >10 and ≤30 | 107.4 (43.2) | 111.7 (39.5) | 103.2 (47.0) | 131.4 (64.3) | 149.1 (73.3) | 114.4 (50.3) |
|  | >30 and ≤60 | 31.8 (25.7) | 27.4 (25.7) | 36.1 (25.5) | 51.0 (48.1) | 49.8 (43.0) | 52.2 (53.5) |
|  | >60 | 7.7 (16.9) | 9.0 (16.2) | 6.5 (17.9) | 32.3 (40.1) | 25.5 (34.1) | 38.8 (45.0) |
| Time-in-bed | | 438.7 (56.0) | 446.4 (59.8) | 431.3 (52.5) | 518.2 (81.7) | 521.0 (79.2) | 515.4 (85.8) |
| **Work from home (WFH)** | | | | | | | |
| Behaviours  and bouts | | Working days | | | Non-working days | | |
|  |  | All workers | BMI <25 | BMI ≥25 | All workers | BMI <25 | BMI ≥25 |
| Sitting | ≤1 | 7.9 (4.3) | 8.9 (4.8) | 7.1 (3.7) | 9.7 (5.3) | 11.9 (5.4) | 7.8 (4.4) |
|  | >1 and ≤5 | 37.5 (17.3) | 41.2 (18.0) | 34.5 (16.3) | 35.9 (14.3) | 39.0 (14.3) | 33.0 (13.9) |
|  | >5 and ≤10 | 56.0 (24.3) | 55.1 (22.7) | 56.8 (25.7) | 53.9 (20.8) | 58.3 (21.6) | 50.0 (19.5) |
|  | >10 and ≤30 | 189.5 (60.0) | 187.1 (60.4) | 191.4 (60.4) | 177.6 (59.6) | 184.6 (62.3) | 171.4 (57.2) |
|  | >30 and ≤60 | 207.2 (62.1) | 206.3 (54.5) | 208.0 (68.3) | 159.8 (78.5) | 143.6 (66.0) | 174.3 (86.6) |
|  | >60 | 271.2 (139.2) | 256.3 (145.4) | 283.1 (134.7) | 199.0 (132.1) | 164.9 (116.2) | 229.5 (139.3) |
| Non-sitting | ≤1 | 12.1 (5.8) | 12.8 (6.0) | 11.5 (5.6) | 11.3 (5.0) | 12.5 (5.5) | 10.3 (4.4) |
|  | >1 and ≤5 | 54.3 (18.1) | 59.6 (20.1) | 50.1 (15.3) | 57.4 (20.7) | 66.1 (21.6) | 49.7 (16.5) |
|  | >5 and ≤10 | 42.1 (19.9) | 46.6 (22.8) | 38.6 (16.6) | 57.6 (26.0) | 64.1 (28.2) | 51.8 (22.7) |
|  | >10 and ≤30 | 67.9 (39.3) | 69.8 (38.3) | 66.5 (40.4) | 102.0 (53.5) | 119.5 (53.5) | 86.5 (49.2) |
|  | >30 and ≤60 | 23.1 (25.9) | 20.8 (22.2) | 24.9 (28.7) | 48.1 (41.1) | 49.3 (34.1) | 47.0 (46.9) |
|  | >60 | 5.8 (15.5) | 3.8 (13.4) | 7.5 (17.0) | 20.6 (32.1) | 16.4 (25.5) | 24.5 (37.0) |
| Time-in-bed | | 465.2 (57.7) | 471.7 (58.7) | 460.0 (57.0) | 506.8 (75.4) | 509.7 (74.3) | 504.3 (77.2) |

In a second step, we examined the results of the detailed EVA matrix to identify occurrences of zero time in the different uninterrupted bouts. After this, we reduced the detailed EVA matrix to fewer categories in which zeros did not occur. Thus, sitting behaviours was categorized in short (≤5 min), moderate (>5 and ≤30 min) and long uninterrupted bouts (>30 min) as done previously (Gupta et al., 2016; Hallman et al., 2018, 2015; Locks et al., 2019, 2018). Non-sitting behaviour was categorized in short (≤5 min) and long uninterrupted bouts (>5 min).

**Reference**

Gupta, N., Hallman, D.M., Mathiassen, S.E., Aadahl, M., Jørgensen, M.B., Holtermann, A., 2016. Are temporal patterns of sitting associated with obesity among blue-collar workers? A cross sectional study using accelerometers. BMC Public Health 16, 1–10. https://doi.org/10.1186/s12889-016-2803-9

Hallman, D.M., Mathiassen, S.E., Gupta, N., Korshøj, M., Holtermann, A., 2015. Differences between work and leisure in temporal patterns of objectively measured physical activity among blue-collar workers. BMC Public Health 15, 976. https://doi.org/10.1186/s12889-015-2339-4

Hallman, D.M., Mathiassen, S.E., Jahncke, H., 2018. Sitting patterns after relocation to activity-based offices: A controlled study of a natural intervention. Prev. Med. (Baltim). 111, 384–390. https://doi.org/10.1016/j.ypmed.2017.11.031

Locks, F., Gupta, N., Hallman, D., Birk Jørgensen, M., Oliveira, A.B., Holtermann, A., 2018. Association between objectively measured static standing and low back pain–a cross-sectional study among blue-collar workers*. Ergonomics 61, 1196–1207. https://doi.org/10.1080/00140139.2018.1455900

Locks, F., Gupta, N., Madeleine, P., Birk Jørgensen, M., Oliveira, A.B., Holtermann, A., 2019. Are accelerometer measures of temporal patterns of static standing associated with lower extremity pain among blue-collar workers? Gait Posture 67, 166–171. https://doi.org/10.1016/j.gaitpost.2018.10.006
