# Supplementary material for: Working at the office or from home during the COVID-19 pandemic: a cross-sectional study of temporal patterns of sitting and non-sitting among normal-weight and overweight Brazilian office workers
Source: J Act Sedentary Sleep Behav. 2023 Dec 5;2:28. doi: 10.1186/s44167-023-00038-0 (PMC11960272; doi:10.1186/s44167-023-00038-0)
Supplement: Supplementary file 2 — Additional file 2: Compositional means of physical behaviours of office workers stratified by gender and age (table S1), mean of the corresponding isometric log-ratio coordinates (Table S2), and the effects of gender and age adjustment variables on each isometric log-ratio (ilr) coordinate (Table S3). [file 44167_2023_38_MOESM2_ESM.docx]

**Additional file 2**

**Additional file 2.** Compositional means of physical behaviours of office workers stratified by gender and age (table S1), mean of the corresponding isometric log-ratio coordinates (Table S2), and the effects of gender and age adjustment variables on each isometric log-ratio (ilr) coordinate (Table S3).

| **Table S1.** Compositional means (SD between workers) in minutes per day and in percent, of sitting, non-sitting and time-in-bed for office workers stratified by gender (women and men) and age (younger [≤ mean age of the sample, i.e. 39.3 years for workers working at the office and 33.5 for workers working from home] and older [> the mean age]). Data are presented for working days and non-working days. | | | | | |
| --- | --- | --- | --- | --- | --- |
| **Gender** | | | | | |
|  | Behaviours | Working days | | Non-working days | |
|  |  | Women | Men | Women | Men |
| Minutes | Sitting short | 51.2 (23.3) | 44.4 (18.7) | 47.7 (21.4) | 47.4 (17.3) |
|  | Sitting moderate | 270.2 (83.6) | 264.6 (82.9) | 216.5 (68.4) | 238.5 (68.7) |
|  | Sitting long | 408.2 (154.5) | 456.0 (144.5) | 322.1 (142.4) | 352.4 (140.9) |
|  | Non-sitting short | 73.0 (24.8) | 70.5 (22.1) | 70.3 (30.4) | 70.4 (22.8) |
|  | Non-sitting long | 171.5 (77.7) | 160.3 (79.1) | 263.0 (114.0) | 229.6 (100.1) |
|  | Time-in-bed | 465.9 (54.6) | 444.2 (60.4) | 520.4 (72.7) | 501.7 (82.0) |
| Percentage | Sitting short | 3.6 (1.6) | 3.1 (1.3) | 3.3 (1.5) | 3.3 (1.2) |
|  | Sitting moderate | 18.8 (5.8) | 18.4 (5.8) | 15.0 (4.7) | 16.6 (4.8) |
|  | Sitting long | 28.3 (10.7) | 31.7 (10.0) | 22.4 (9.9) | 24.5 (9.8) |
|  | Non-sitting short | 5.1 (1.7) | 4.9 (1.5) | 4.9 (2.1) | 4.9 (1.6) |
|  | Non-sitting long | 11.9 (5.4) | 11.1 (5.5) | 18.3 (7.9) | 15.9 (6.9) |
|  | Time-in-bed | 32.4 (3.8) | 30.8 (4.2) | 36.1 (5.1) | 34.8 (5.7) |
| **Age** | | | | | |
|  | Behaviours | Working days | | Non-working days | |
|  |  | Younger | Older | Younger | Older |
| Minutes | Sitting short | 44.6 (19.0) | 53.3 (23.9) | 45.3 (17.3) | 51.0 (21.9) |
|  | Sitting moderate | 260.2 (82.0) | 279.3 (84.1) | 232.7 (75.5) | 219.1 (57.5) |
|  | Sitting long | 464.1 (153.1) | 378.9 (133.0) | 351.4 (134.2) | 314.7 (151.9) |
|  | Non-sitting short | 68.3 (22.7) | 77.5 (23.8) | 70.0 (26.3) | 70.9 (27.8) |
|  | Non-sitting long | 146.7 (76.1) | 197.2 (72.0) | 230.5 (108.3) | 271.5 (104.3) |
|  | Time-in-bed | 456.2 (55.0) | 453.8 (63.8) | 510.1 (76.0) | 512.8 (81.1) |
| Percentage | Sitting short | 3.1 (1.3) | 3.7 (1.7) | 3.1 (1.2) | 3.5 (1.5) |
|  | Sitting moderate | 18.1 (5.7) | 19.4 (5.8) | 16.2 (5.2) | 15.2 (4.0) |
|  | Sitting long | 32.2 (10.6) | 26.3 (9.2) | 24.4 (9.3) | 21.9 (10.6) |
|  | Non-sitting short | 4.7 (1.6) | 5.4 (1.6) | 4.9 (1.8) | 4.9 (1.9) |
|  | Non-sitting long | 10.2 (5.3) | 13.7 (5.0) | 16.0 (7.5) | 18.9 (7.2) |
|  | Time-in-bed | 31.7 (3.8) | 31.5 (4.4) | 35.4 (5.3) | 35.6 (5.6) |

| **Table S2.** Mean (with SD between workers) of the isometric log-ratio (ilr) coordinates of office workers stratified by gender (women and men) and age (younger [≤ mean age of the sample, i.e. 39.3 years for workers working at the office and 33.5 for workers working from home] and older [> the mean age]). Data are presented for working days and non-working days. | | | | |
| --- | --- | --- | --- | --- |
| **Gender** | | | | |
| ilr-coordinates | Working days | | Non-working days | |
|  | Women | Men | Women | Men |
| ilr_1_: time-in-bed/awake | 1.12 (0.25) | 1.09 (0.26) | 1.25 (0.26) | 1.19 (0.27) |
| ilr_2_: sitting/non-sitting | 0.53 (0.45) | 0.59 (0.41) | 0.10 (0.44) | 0.25 (0.40) |
| ilr_3_: sitting short/moderate+long | -1.55 (0.44) | -1.70 (0.38) | -1.42 (0.52) | -1.48 (0.40) |
| ilr_4_: sitting moderate/long | -0.26 (0.55) | -0.38 (0.44) | -0.23 (0.48) | -0.25 (0.46) |
| ilr_5_: non-sitting short/long | -0.55 (0.43) | -0.50 (0.42) | -0.93 (0.41) | -0.80 (0.40) |
| **Age** | | | | |
| ilr-coordinates | Working days | | Non-working days | |
|  | Younger | Older | Younger | Older |
| ilr_1_: time-in-bed/awake | 1.15 (0.26) | 1.04 (0.24) | 1.23 (0.27) | 1.21 (0.26) |
| ilr_2_: sitting/non-sitting | 0.66 (0.46) | 0.39 (0.30) | 0.23 (0.42) | 0.09 (0.41) |
| ilr_3_: sitting short/moderate+long | -1.69 (0.41) | -1.51 (0.42) | -1.51 (0.44) | -1.35 (0.48) |
| ilr_4_: sitting moderate/long | -0.39 (0.52) | -0.21 (0.45) | -0.27 (0.47) | -0.19 (0.47) |
| ilr_5_: non-sitting short/long | -0.45 (0.45) | -0.64 (0.35) | -0.81 (0.43) | -0.94 (0.36) |

| **Table S3.** Effects of gender (women *vs.* men) and age (younger [≤ mean age of the sample, i.e. 39.3 years for workers working at the office and 33.5 for workers working from home] and older [> the mean age]) adjustment variables on each isometric log-ratio (ilr) coordinate. The table shows effect size (partial eta squared [η_p_^2^]), F-statistics, and *p*-value. | | | | | | |
| --- | --- | --- | --- | --- | --- | --- |
| **Working days** | | | | | | |
|  | Women *vs.* men | | | Younger *vs.* older | | |
| ilr-coordinates | η_p_^2^ | F | *p*-value | η_p_^2^ | F | *p*-value |
| ilr_1_: time-in-bed/awake | <0.01 | 0.24 | 0.62 | **0.04** | **4.57** | **0.03** |
| ilr_2_: sitting/non-sitting | 0.01 | 1.13 | 0.29 | **0.10** | **11.82** | **0.001** |
| ilr_3_: sitting short/moderate+long | **0.04** | **4.57** | **0.03** | **0.05** | **5.58** | **0.02** |
| ilr_4_: sitting moderate/long | 0.02 | 2.35 | 0.13 | 0.03 | 2.88 | 0.09 |
| ilr_5_: non-sitting short/long | 0.01 | 0.64 | 0.42 | **0.04** | **4.71** | **0.03** |
| **Non-working days** | | | | | | |
|  | Women *vs.* men | | | Younger *vs.* older | | |
| ilr-coordinates | η_p_^2^ | F | *p*-value | η_p_^2^ | F | *p*-value |
| ilr_1_: time-in-bed/awake | 0.02 | 1.82 | 0.18 | <0.01 | 0.23 | 0.63 |
| ilr_2_: sitting/non-sitting | 0.03 | 3.06 | 0.08 | **0.03** | **3.86** | **0.05** |
| ilr_3_: sitting short/moderate+long | <0.01 | 0.22 | 0.64 | **0.04** | **4.69** | **0.03** |
| ilr_4_: sitting moderate/long | <0.01 | <0.01 | 0.96 | 0.01 | 0.90 | 0.34 |
| ilr_5_: non-sitting short/long | 0.03 | 3.40 | 0.07 | 0.02 | 2.33 | 0.13 |
| Results with *p*-value < 0.05 are shown in bold. | | | | | | |
